# Bilateral middle ear cholesteatoma in children: A single-center retrospective study

**DOI:** 10.1016/j.bjorl.2025.101671

**Published:** 2025-07-03

**Authors:** Xiaoxu Wang, Lining Guo, Enxia Tian, Min Chen, Wei Liu, Xin Ni, Jie Zhang

**Affiliations:** a Capital Medical University, Beijing Children′s Hospital, National Center for Children’s Health, National Key Clinical Specialty, Department of Otorhinolaryngology Head and Neck Surgery, Beijing, China; b Beijing Key Laboratory for Pediatric Diseases of Otolaryngology Head and Neck Surgery, Beijing, China; c Children's Hospital Affiliated to Zhengzhou University, Henan Children's Hospital, Zhengzhou Children’s Hospital, Department of Otorhinolaryngology Head and Neck Surgery, Henan Province Engineering Research Center of Early Diagnosis and Precise Treatment of Sleep Disordered Breathing, Zhengzhou, China

**Keywords:** Middle ear cholesteatoma, Bilateral, Children, Clinical characteristics, Treatment pathway

## Abstract

**Objectives:**

To analyze the clinical characteristics of bilateral cholesteatoma in children and summarize the clinical treatment pathway.

**Methods:**

In this retrospective cohort study, we analyzed the records of children with bilateral middle ear cholesteatoma who underwent surgery in our department between 2016 and 2023. The clinical characteristics, treatment and prognosis were analyzed.

**Results:**

The records of 12-children (24-ears, 4girls and 8-boys) were reviewed. The mean age was 6.5 ± 2.6-years. Four children had Congenital Cholesteatoma (CC) in one ear and Acquired Cholesteatoma (AC) in the other ear and 8-children had AC in both ears. The onset time and lesion degree of the bilateral ears are different. Nine children had maxillary developmental deformities (75%). One child underwent simultaneous binaural surgery, and 11 underwent staged surgery, with a median interval of 3-months between surgeries. The follow-up period was 4.28 ± 2.58-years. No postoperative complications. Six children (7-ears) underwent revision surgery due to recurrence. There was no significant difference in hearing results between pure-tone average of preoperative (41.23 ± 16.50 dB) and postoperative (37.79 ± 14.42 dB) (p = 0.291, *t* = 1.082). There was no significant difference in postoperative hearing between CC and AC (p = 0.355, *F* = 4.268). There were significant differences in postoperation hearing among different surgical methods (p = 0.006, H = 12.630). There were significant differences in postoperation hearing among different ossiculoplasty methods (p = 0.001, H = 17.590).

**Conclusions:**

In our cohort, the average age at diagnosis of bilateral cholesteatoma in children was notably young, with a high proportion presenting concurrent cleft palate deformities. Staged surgical intervention prioritizing the more severely affected ear was adopted for bilateral cases. Furthermore, long-term follow-up is necessary due to the observed high recurrence rate in this population. A management flowchart has been proposed based on our therapeutic experience.

**Level of evidence:**

Level 4.

## Highlights

• The incidence of cleft palate malformation in BMEC is high.
• Staging bilateral surgery is preferred and surgery on the worse side first.
• Simultaneous bilateral surgery in children should be carefully considered.
• Postoperative hearing showed no significant difference between CC and AC in BMEC.
• BMEC has a high recurrence rate after operation and require a long-term follow-up.

## Introduction

Cholesteatoma is a mass formed by the keratinizing, squamous epithelium in the tympanic cavity and/or mastoid and subepithelial connective tissue, and progressive accumulation of keratin debris.^1^ Although cholesteatomas are not true tumors, they can proliferate, and erode and destroy the structure of the middle ear, resulting in hearing loss and serious intracranial and extracranial complications. The annual incidence of middle ear cholesteatoma is approximately 12.6 per 100,000 in adults, and 3 per 100,000 in children.^2^ Among them, the incidence of binaural disease is about 4.4 %–17.1 %.^3^, ^4^

In children, Bilateral Middle Ear Cholesteatoma (BMEC) can cause bilateral hearing loss and requires long-term medical treatment. It can have a serious effect on children’s learning and social adaptation and may affect the speech development of young children. The treatment strategy of BMEC needs more consideration. Published literature on BMEC is limited to a few small series and case reports published during previous decades.^5^, ^6^, ^7^, ^8^ Currently no standard protocols are available for the management of BMEC in children. Retaining or improving hearing and reducing the recurrence rate make management challenging. In this paper, we review previous cases of BMEC treated at our institution and propose a treatment algorithm based on our experience to improve the understanding of the disease and provide guidance for clinical diagnosis and treatment.

## Methods

### Study design

A retrospective chart and surgical video review were completed for children with BC who underwent surgery at the Otolaryngology Department of Beijing Children’s Hospital between 2016 and 2023. The inclusion criteria were age ≤18-years, completed the first surgical treatment of BMEC in our department, and were followed up for more than 1-year. Patients with incomplete medical records were excluded. All cholesteatoma diagnoses were pathologically confirmed. The data included details on patient demographics, medical history, physical examination, imaging, extent of lesions, degree of mastoid development, audiometry, treatment and follow-up, and outcome.

**Fig. 1:**
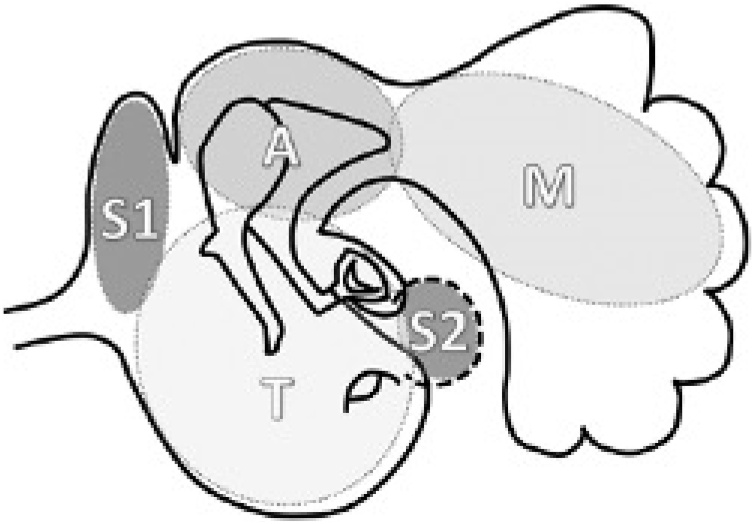
Divisions of the middle ear space using the STAM system.^1^ S, The difficult access sites (includes S1, the Supratubal recess and S2, the Sinus tympani); T, Tympanic cavity; A, Attic; M, Mastoid.

The classification of cholesteatoma is divided into CC and AC according to the definition of the European Academy of Otology and Neurotology and Japan Otological Society (EAONO/JOS).^1^ The extent of cholesteatoma was described using the STAM staging system also proposed by EAONO/JOS.^1^ The middle ear and mastoid space were divided into four sites: difficult to access Sites (S), the Tympanic cavity (T), the Attic (A) and the Mastoid (M). The difficult access Sites (S) included S1, the supratubal recess, and S2, the sinus tympani (Fig. 1). The degree of mastoid development was classified into three types according to the finding on Computed Tomography (CT) of the temporal bone CT: pneumatic type (numerous large air cells, with high translucency and honeycomb shape); diploetic type (part of the mastoid is pneumatized, with small and dense air cells); and sclerotic type (the mastoid is composed of compact bone). According to the age and degree of cooperation of the children, hearing was tested using pure-tone audiometry or game audiometry. The Pure-Tone Average (PTA) was defined as the average of hearing sensitivity thresholds to pure-tone signals at 500, 1000, 2000, and 4000 Hz.

The cholesteatoma resection procedures included Canal Wall Up (CWU), Canal Wall Down (CWD), and Transcanal Endoscopic Ear Surgery (TEES). The specific surgical method is selected based on the extent of the lesion. The ossiculoplasty were divided into five categories according to the different condition: (1) With Partial Ossicular Replacement Prosthesis (PORP), (2) With Total Ossicular Replacement Prosthesis (TORP), (3) With tragus cartilage, (4) With complete ossicular chain and no need for ossiculoplasty, and (5) Without ossiculoplasty for various special reasons.

The main outcome indicators were recurrence of cholesteatoma, and postoperative long-term hearing results. The diagnosis of recurrence was based on clinical examination, otoscopy findings, or performance of Diffusion-Weighted Imaging Magnetic Resonance Imaging (DWI MRI) after surgery. The postoperative long-term hearing results were determined at the most recent follow-up (at least 1-year after the surgery).

This study was approved by the Research Ethics Board of Medical Ethics Committee, Beijing Children’s Hospital Affiliated to Capital Medical University and the study was conducted in accordance with the principles of the Declaration of Helsinki and its later amendments. The research ethics board waived the requirement for written informed consent owing to the retrospective study design.

### Statistical analysis

Statistical analysis was performed using SPSS 27. Continuous variables were reported as means and standard deviations or medians and ranges, and categorical variables were reported as frequencies and percentages. The normality test used the Shapiro-Wilk test. Paired *t*-test was used for hearing results before and after surgery. Based on the non-normal distribution of the data, Kruskal Wallis was used to compare the prognosis of hearing with different conditions; p-values <0.05 were considered statistically significant.

## Results

Between 2016 and 2023, a total of 320 pediatric with cholesteatoma were treated in our department, including 12 cases of BMEC with 4 girls and 8 boys. Their characteristics are shown in Table 1. The mean age at diagnosis was 6.5 ± 2.6-years (rang from 2.5 to 9.8), with only one child aged younger than 3-years. The mean duration of follow-up was 4.28 ± 2.58-years.

**Table 1:** Demographic data and medical information of 12 children with bilateral middle ear cholesteatoma.

ID	Sex	Age^a^ (years)	Side	Classification	Course^b^ (months)	Palate development	Mastoid development	Extension	Per-operative PTA (dB)	Binaural surgery interval (months)	Surgery	Postoperative recurrence and time	Total number of operations	Ossiculoplasty	Post-operative PTA (dB)	Follow up time (years)
1	B	9.5	R	AC	84	Cleft palate	Diploetic	STAM	62.5	36	CWU	Yes (0.8 years)	3	Without	46.25	5
			L	AC	84		Diploetic	STAM	62.5		CWD	NO		Cartilage	40.0	
2	B	3.3	L	AC	1	Cleft lip and palate	Diploetic	STA	63.75	1	CWU	Yes (2 years)	3	Without	62.5	9
			R	AC	1		Diploetic	STA	32.5		CWU	NO		TORP	38.75	
3	B	7.0	L	AC	24	Without	Pneumatic	STAM	36.25	4	CWU	NO	3	PORP	31.25	1.3
			R	AC	24		Pneumatic	STAM	38.75		CWU	NO		PORP	30.0	
4	B	3.4	L	AC	2	Occult submucosal cleft palate	Diploetic	STAM	20.0	8	CWU	NO	3	No Need	16.25	8.8
			R	AC	2		Diploetic	STA	30.0		CWU	Yes (2.5 years)		Cartilage	50	
5	G	9.5	R	AC	24	Occult submucosal cleft palate	Sclerotic	STAM	65.0	36	CWU	Yes (3 years)	3	Without	52.0	4
			L	AC	0		Sclerotic	STA	10.0		CWU	NO		No Need	10.0	
6	G	9.8	L	AC	12	Without	Diploetic	STAM	57.5	13	CWU	Yes (0.9 years)	3	Without	65.0	1.7
			R	AC	24		Diploetic	STAM	41.25		CWD	NO		Cartilage	31.25	
7	B	7.4	L	CC	6	Without	Diploetic	T	10.0	0	TEES	NO	2	No Need	31.25	5
			R	AC	6		Pneumatic	STAM	35.0		CWD	NO		TORP	37.5	
8	G	5.8	R	AC	12	Cleft palate	Diploetic	STAM	61.25	16	CWD	NO	2	Cartilage	51.25	5.3
			L	CC	0		Diploetic	STA	55.0		CWU	NO		PORP	26.25	
9	B	2.5	L	CC	3	Occult submucosal cleft palate	Diploetic	T	35.0	1	CWU	Yes (1.3 years)	4	TORP	36.25	4.4
			R	AC	3		Diploetic	STAM	40.0		CWU	Yes (1.5 years)		TORP	55.0	
10	G	9.2	R	AC	1	Occult submucosal cleft palate	Diploetic	STAM	37.25	2	CWD	NO	2	Without	46.25	2
			L	AC	0		Diploetic	STA	37.5		CWD	NO		Cartilage	45.0	
11	B	4.8	R	AC	12	Occult submucosal cleft palate	Diploetic	STAM	56.25	3	CWD	NO	2	PORP	12.5	3
			L	CC	0.3		Diploetic	TAM	21.25		CWU	NO		PORP	32.5	
12	B	5.9	L	AC	36	Occult submucosal cleft palate	Diploetic	STAM	35.0	2	CWD	NO	2	TORP	35.0	1.8
			R	AC	36		Diploetic	STAM	46.25		CWD	NO		PORP	25.0	

AC, Acquired Cholesteatoma; CC, Congenital Cholesteatoma; S, The difficult access sites; T, Tympanic cavity; A, Attic; M, Mastoid; CWU, Canal Wall Up; CWD, Canal Wall Down; TEES, Transcanal Endoscopic Ear Surgery; PORP, Partial Ossicular Replacement Prosthesis; TORP, Total Ossicular Replacement Prosthesis; PTA, Pure-Tone Average.

a Age: Age at first diagnosis of middle ear cholesteatoma.

b Course: Duration of ear symptoms.

**Fig. 2:**
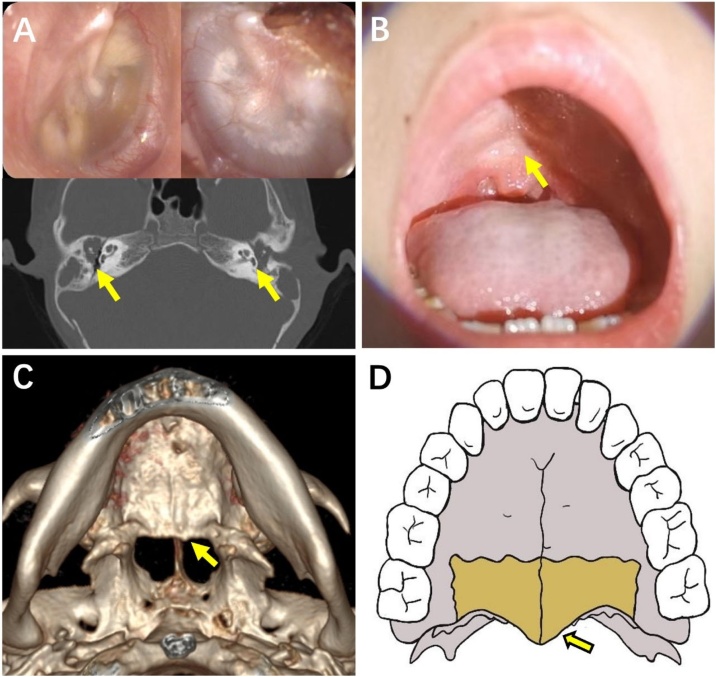
Case 12. (A) Otoscope and CT scan on temporal bone. (B) An inverted V-shaped depression at the junction of the soft palate and the hard palate. (C) The three-dimensional reconstruction of the maxillary CT scan confirmed the absence of posterior nasal spine. (D) The hand drawing shows a complete posterior nasal spine structure.

Four children had CC in one ear and AC in the other ear and 8 children had AC in both ears. The course of disease in the bilateral ears of the same child is different, but there is no significant difference statistically (p = 0.264, *t* = 1.178). Three children (Cases 6, 10, and 11) had a different course of disease in bilateral ears and two children (Cases 5 and 8) had unilateral middle ear cholesteatoma at the time of their first visit and subsequently developed a cholesteatoma in the contralateral ear. The extent of the lesions differed in bilateral ears in 6 children (Cases 4, 7, 8, 9, 10 and 11) were different. Nine children had maxillary developmental deformities (75%) (cleft palate in 3 cases and occult submucosal cleft palate in 6 cases), all of nine children were accompanied by diploetic or sclerotic type mastoid. Fig. 2 shows the details of a BMEC in a child (Case 12) with occult submucosal cleft palate. There was only 1 case of pneumatolytic type in bilateral mastoid.

The surgical treatment varied according to the severity of the lesion. The specific surgical methods and ossiculoplasty are shown in Table 1. Only one child (Case 7) underwent simultaneous binaural operation, and the other 11 children underwent staged surgery. The median interval between the first and second surgery was 3-months (range: 1–12 months). Six children (7-ears) (Cases 1, 2, 4, 5, 6, and 9) experienced a recurrence. In the revision operation due to recurrence, 6-ears underwent CWD and one ear underwent CWU surgery. The ossiculoplasty may be performed in the first or second-look stage. The final procedure method used for ossiculoplasty is shown in Table 1. The mean number of operations per patient was 2.67 ± 0.65. The mean preoperative PTA was 41.23 ± 16.50 dB, and the mean long-term postoperative PTA was 37.79 ± 14.42 dB. There was no significant difference in hearing results between preoperative and postoperative patients (p = 0.291, *t* = 1.082). No postoperative complications such as sensorineural deafness or facial paralysis were observed in any of the children.

There was no significant difference in postoperative hearing between CC and AC (p = 0.355, F = 4.268). There were significant differences in postoperation hearing among different surgical methods (p = 0.006, H = 12.630). In pairwise comparison, there were significant differences in postoperative hearing between the CWU group and the group undergoing recurrent surgery (p = 0.003). There was no significant difference between the other groups. There were significant differences in postoperation hearing among different ossiculoplasty methods (p = 0.001, H = 17.590). In a pairwise comparisons, there was significant difference in hearing outcomes between the without ossiculoplasty group and PORP group (p = 0.006). There was significant difference between the without ossiculoplasty group and no need ossiculoplasty group (p = 0.013). And among the other groups there were no significant differences (Table 2).

**Table 2:** The relationship between long-term postoperative hearing prognosis and classification, surgical methods and ossicloplasty methods.

	Category	n	Postoperative PTA (x ± S dB)	p	Test statistics
Classification	AC	20	39.04 ± 15.46	0.355	F = 4.268
	CC	4	31.56 ± 4.13		
Surgery	CWU	7	26.43 ± 9.99	0.006	H = 12.630
	CWD	9	35.97 ± 11.90		
	TEES	1	31.25		
	Recurrence surgery	7	52.43 ± 9.76		
Ossiculoplasty	PORP	6	26.25 ± 7.33	0.001	H = 17.590
	TORP	5	40.50 ± 8.20		
	Cartilage	5	43.50 ± 8.16		
	No need	3	19.17 ± 10.92		
	Without	5	54.40 ± 8.90		
Overall	‒	24	37.79 ± 14.42	‒	‒

AC, Acquired cholesteatoma; CC, Congenital Cholesteatoma; CWU, Canal Wall Up; CWD; Canal Wall Down; TEES, Transcanal Endoscopic Ear Surgery; PORP, Partial Ossicular Replacement Prosthesis; TORP, Total Ossicular Replacement Prosthesis; PTA, Pure-Tone Average.

## Discussion

This paper reports 12 cases of BMEC in children. To our knowledge, this is the largest case series on children to date with complete long-term follow-up data. This study revealed that the age at diagnosis was relatively young, with a median age of 6.5-years (considering the delay in diagnosis, the age of onset is likely to have been younger). In addition, bilateral ears had different course, different degrees of lesion, and lesions at different locations. The BMEC in this study included both congenital and acquired, and the pathogenic mechanisms are likely to have been multifactorial. In our cases, palatal malformations, including cleft palate and occult submucosal cleft palate, were important risk factors. According to previous studies, children with cleft palate are prone to middle ear cholesteatoma and otitis media because of abnormalities in Eustachian tube anatomy and function, viscosity of middle ear effusion, and frequency of middle ear infection.^9^ The incidence of cholesteatoma in children with cleft palate is between 1.5% and 5.9%, which is 200 times higher than that in the general population.^10^, ^11^, ^12^ It is worth paying attention to that children with occult submucosal cleft palate are also susceptible to BMEC. In this study, 3 children had cleft palate, and 6 children had occult submucosal cleft palate. Occult submucosal cleft palate is caused by the defect of a part of the posterior nasal spine of the hard palate.^13^ Physical examination shows an inverted V-shaped depression at the junction of the soft palate and the hard palate when making an “ah” sound (Fig. 2B), with or without speech impediments. The diagnosis of occult submucosal cleft palate in children is often overlooked because the defect is not easily detected. The middle ear problems associated with occult submucosal cleft palate have not been reported. The palate should be carefully monitored during physical examination. If signs of occult submucosal cleft palate are present, attention should be paid to middle ear problems in the early stage. In addition, habitual sniffing extraction caused by patulous eustachian tube may be related to the pathogenesis of BMEC^14^, ^15^ owing to the presence of total closing failure, with an intermittent free transfer of respiratory pressure to the middle ear, the sniffing procedure maintains a negative intratympanic pressure, gradually forming an attic retraction pocket.^16^ However, in our children, no patulous eustachian tube was found. Additional etiologies of BMEC requires analysis of a larger samples.

In the case of binaural lesions, repeated surgeries and bilateral hearing loss can have a significant impact on children’s development, and complete removal of the lesion and protection of hearing are particularly important. In terms of the surgical treatment strategy, the first choice is between simultaneous or staged surgery. In the traditional experience, bilateral chronic middle ear disease is typically performed by staging surgery for the risk of iatrogenic bilateral sensorineural hearing loss, bilateral facial nerve injury, and temporary bilateral hearing loss during the recovery period in simultaneous binaural surgery. However, there are also arguments for the advantages of simultaneous binaural surgery, such as reduces the cost of surgery and improves convenience for patients and surgeons. We tend to believe that staging surgery is preferred. But if you are quite confident in the surgery for the first ear won’t be injured, simultaneous binaural surgery can be considered. Of the 12 children in our group, 11 underwent binaural staged surgeries and only one underwent simultaneous surgery. The simultaneous surgery was performed because one of his first ear is CC with a very limited small lesion and without any structural damage, the surgery of the ear went very smoothly and quick under otoscopy. In all staged surgeries, the more severe side is selected first. For the Klemens et al.^17^ suggested another situation that surgeons should consider before surgery of bilateral: Are the anatomical and pathological conditions of the first ear sufficiently unusual or difficult that it is safer, for the patient, to address the second ear while all the relationships are fresh in the surgeon’s mind?. We haven't encountered this unusual situation, but it's worth considering. In summary, simultaneous binaural surgery in children requires more caution and certainty.

In this study, there was no statistical difference in hearing changes before and after surgery, but the PTA of long-term hearing after surgery was better than that before surgery. And there was no significant difference in hearing outcomes between CC and AC. The hearing outcomes were worse in the group that did not undergo ossiculoplasty and worse in the group that underwent recurrent surgery. And there was no significant difference in hearing prognosis between CWU, CWD and TEES groups. There was No statistical difference between PORP, TORP, Cartilage and did not need ossiculoplasty groups. However, the limitation is that the sample size of each group is small. The specific surgical method and the type of ossiculoplasty need to be personalized according to the patient's condition and intraoperative states. Other factors in the actual situation may also lead to unsatisfactory postoperative hearing recovery, such as extensive lesion destruction, congenital eustachian tube dysfunction, poor middle ear ventilation or postoperative ossicle displacement. Early use of hearing aids should be considered in children with poor hearing in both ears (moderate or higher hearing loss) after stabilization of the ear condition after surgery.

**Fig. 3:**
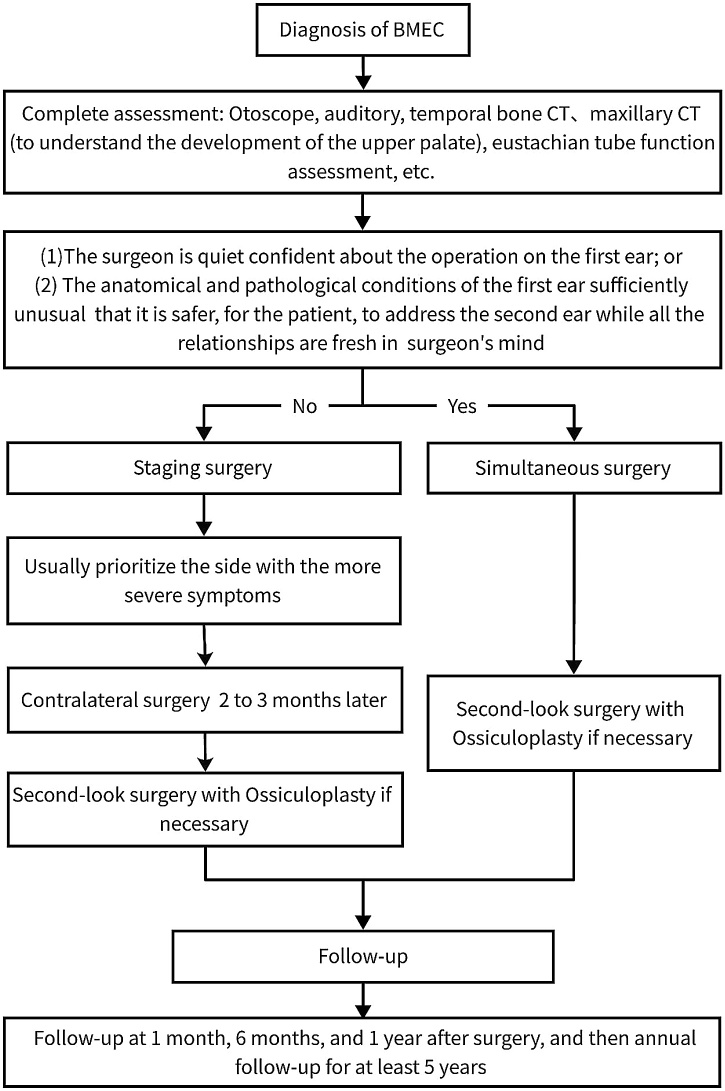
Flow chart of clinical treatment for BMEC in children. BMEC: bilateral middle ear cholesteatoma.

The high recurrence rate of the surgeries (7/24, 29.2%) shown in this study may be related to the presence of certain risk factors in these children. In this study, the recurrence time was 0.8‒3-years after surgery and the longest follow-up was 9-years. About the postoperative follow-up, International Pediatric Oncology Group (IPOG) guidelines^18^ recommend a minimum of 5-years. Most of the children had no interruption in follow-up. We recommend that children with BMEC be followed-up for more than 5-years or even for life. Based on our experience, we summarized a treatment flow chart for BMECs (Fig. 3).

## Conclusion

In our cohort, BMEC presented at a significantly younger age and exhibited a high prevalence of concomitant palatal cleft anomalies or occult submucous cleft palates, necessitating particular clinical vigilance in this pediatric population. A staged bilateral surgical intervention was prioritized in most cases, with the more symptomatic ear addressed first. Furthermore, given the elevated recurrence risk in these patients, long-term follow-up surveillance is imperative.

## ORCID ID

Xiaoxu Wang: 0000-0002-2329-8730

Lining Guo: 0000-0002-3660-4864

Enxia Tian: 0009-0002-7199-5394

Min Chen: 0000-0001-8935-5606

Wei Liu: 0000-0002-1927-5499

Xin Ni: 0000-0003-0857-8512

Jie Zhang: 0000-0002-1621-7779

## Funding

The authors declare that no funds, grants, or other support were received during the preparation of this manuscript.

## Declaration of competing interest

The authors declare no have conflicts of interest.
